# Enzyme histochemical characterization of orbital glands in fetuses of Indian buffalo (*Bubalus bubalis*)

**DOI:** 10.7717/peerj.15196

**Published:** 2023-04-10

**Authors:** Mahendra Pratap Singh Tomar, Neelam Bansal

**Affiliations:** 1 Veterinary Anatomy, N.T.R. College of Veterinary Science, Gannavaram, Vijayawada, Andhra Pradesh, India; 2 Veterinary Anatomy, Guru Angad Dev Veterinary and Animal Sciences University, Ludhiana, Punjab, India

**Keywords:** Buffalo fetus, Enzyme histochemistry, Harderian gland, Lacrimal gland, Orbital gland, Tear production

## Abstract

**Background:**

The orbital glands, *viz*. lacrimal gland, superficial and deep gland of third eyelid (LG, SGT and HG), are important for normal eye functions. These glands have different functions in various animals. The information about the enzyme histochemical nature of prenatal orbital glands in Indian buffalo seems to be unavailable. Therefore, the study was planned on orbital glands of six full term recently died fetuses from animals with dystocia.

**Methods:**

The frozen sections of all these glands were subjected to standard localization protocols for Alkaline Phosphatase (AKPase), Glucose 6 phosphatase (G-6-Pase), Lactate dehydrogenase (LDH), Succinate dehydrogenase (SDH), Glucose 6 phosphate dehydrogenase (G-6-PD), Nicotinamide Adenine Dinucleotide Hydrogen Diaphorase (NADHD), Nicotinamide Adenine Dinucleotide Phosphate Hydrogen diaphorase (NADPHD), Dihydroxy phenylalanine oxidase (DOPA-O), Tyrosinase, non-specific esterase (NSE) and Carbonic anhydrase (CAse).

**Results:**

The results revealed a mixed spectrum of reaction for the above enzymes in LG, SGT and HG which ranged from moderate (for LDH in SGT) to intense (for most of the enzymes in all three glands). However, DOPA-O, Tyrosinase and CAse did not show any reaction. From the present study, it can be postulated that the orbital glands of fetus have a high activity of metabolism as it has many developmental and functional activities which were mediated with the higher activity of the enzymes involved.

## Introduction

Enzyme histochemistry is a sensitive dynamic technique where visualization is accomplished with an insoluble dye product which mirrors even early metabolic imbalances in an organism. It can be combined with the advantage of histotopographic enzyme localization to understand the entire mechanism (Meier-Ruge & Bruder, 2008). In terms of fuel metabolism, the fetus and its mother coexist in a complex, dynamic, and yet fascinating relationship. Although the fetus is principally dependent on maternal resources to meet its high nutritional needs, it is nonetheless capable of some independent metabolism. Maternal anabolism causes storage during the first half of pregnancy, but exponential fetal growth occurs during the second half, causing maternal stores to be mobilised. Although glucose is mostly used as a substrate for energy generation, fetuses have the ability to utilise other fuels, such as lactate, ketoacids, amino acids, fatty acids, and glycogen, in certain situations (Rao, Shashidhar & Ashok, 2013). During prenatal development, a rapid proliferation of the cells, synthesis of fetal cell components and intercellular substances require high energy supply therefore a rhythmic activity (periodic stimulation and inactivation/inhibition) of different enzymes is required to regulate them (Nelson & Cox, 2004). During cell division, cell differentiation and remodeling of the tissue, the energy is supplied through active exchange of phosphate between molecules. The supply of the energy is meant through the aerobic breakdown of sugar molecules and the stored fat in the fetal body (Rao, Shashidhar & Ashok, 2013).

The orbital glands, *viz*., lacrimal gland, superficial gland (*Glandula Profunda*) and deep gland (Harderian glands) of the third eyelid (LG, SGT and HG), are important for normal eye functions. Among these glands, the superficial gland (SGT) is observed in mammals with a third eyelid (Nawrot et al., 2013). The glands of third eyelids are located in the medial canthus, while the LG is present at dorso-lateral aspect of eye within the orbit. The deep gland of third eyelid is predominantly a cluster of mucin secreting tissue, whereas the LG contributes as part of the lacrimal apparatus and produces the aqueous layer of the tear film (Payne, 1994; Sahadkhast et al., 2008). In cattle, rodents and birds, the HG is responsible for secretion of the aqueous layer of the tear film, which moisturizes the cornea (Davidson & Kuonen, 2004). The functions of HG vary among animal species as a site for immunoglobulins production in birds (Van Ginkel et al., 2012) or performs a photoprotective role in rodents a part of a photoreceptive pineal-related axis or on other hand acts as a source of pheromones and growth factors but it lubricates the eye and the third eyelid (TE) in mammals (Chieffi et al., 1996). The SGT and LG in cattle are predominantly mucoserous in nature and help in protection of ocular surface (conjunctiva and cornea). Presence of antibacterial factors and its involvement in the regeneration of the damaged cornea were also reported in aqueous layer of tear film (Nawrot & Dziegiel, 2008). The histological structure of orbital glands was reported in many species of mammals such as one humped camel, goat, and donkey (Alsafy, 2010)_,_ sheep (Gargiulo et al., 2000), porcine (Henker et al., 2013)_,_ American bison (Pinard et al., 2003) and European bison (Nawrot et al., 2015).

The available literature revealed a few information on the enzyme histochemical characterization of these glands in animals. However, the enzymatic activity specially of lysosomal and amylase types was reported in tears (Anderson & Leopold, 1979). Similarly, the presence of lactate dehydrogenase, peroxidase and amylase enzymes were also observed in the tear, lacrimal gland fluid and the tissue of rat (Thorig, Van Haeringen & Wijngaards, 1984). Some enzyme histochemical studies were also observed in extraocular muscles (Tomar & Bansal, 2017) and eyelids (Tomar & Bansal, 2019) of buffalo fetuses but the research related to the enzyme histochemistry of orbital glands in Indian buffalo were not found as per best of our knowledge. Therefore, the study was planned on the histochemical localization of various enzymes in orbital glands of fetuses in Indian buffalo which may be the first ever reports on enzyme histochemical localization of glands of buffalo fetuses.

## Materials and Methods

The enzyme histochemical characterization of orbital glands was carried out on the lacrimal gland; superficial and deep gland of third eyelid obtained from total six buffalo fetuses immediately after their natural deaths or dam’s death. These fetuses were collected from the buffaloes presented either at Teaching Veterinary Clinical Complex of Guru Angad Dev Veterinary and Animal Sciences University (GADVASU) for release of dystocia or for necropsy at postmortem hall of Guru Angad Dev Veterinary and Animal Sciences University (GADVASU), Ludhiana, India or from the local slaughter houses (in emergency slaughters). None of these fetuses was collected in live condition therefore, the ethical permission was not required. They were ranged from 89.0 cm Crown Vertebral Rump Length (CVRL) to 94.0 cm CVRL (274 to 286 days of gestation). The estimation of the gestational age was done with following formula (Soliman, 1975):

$\rm Y= 73.544+2.256\, X \, (CVRL \gt 20\, cm)$

where, Y is the age in days and X is CVRL in centimeters. Fresh unfixed tissues from lacrimal gland, superficial and deep gland of third eyelids in prenatal buffalo were collected and placed in deep refrigerator under −20 °C. The Leica cryostat microtome at −20 °C was used to get cryostat sections of 10 µm thicknesses and the sections were obtained on glass slides. The glands of right and left sides were considered separate specimens and from each specimen, at least two slides with 2–3 section on each were subjected for investigation. These sections were then incubated with appropriate substrates for the demonstration of alkaline phosphatase, glucose-6-phosphatases (Barka & Anderson, 1963; Bancroft, 2002), lactate dehydrogenase, succinate dehydrogenase, glucose-6-phosphate dehydrogenase (Bancroft, 2002; Pearse, 1972), NADH & NADPH diaphorases (Pearse, 1972) DOPA oxidase, tyrosinase (Bancroft, 2002; Roy, 2002) and carbonic anhydrase (Roy, 2002; Sugimoto et al., 1988). The positive and negative controls were also carried out wherever possible. All the sections were observed under the compound light microscope at 100 and 400× magnification. These sections were viewed on screen with Nikon eclipse 80i microscope with inbuilt camera (the light exposures were set to auto mode) and photomicrographs representing the best field were captured. These images for all enzymes were examined with naked eye (on computer screen) to see the difference in color intensity and was given grades if they differ distinctly *viz*., -- for negative, + for weak positive, ++ for moderate positive and +++ for strong positive reactions. The same grades were allotted to each image until the distinct differences were seen among the intensity of reactions.

## Results

**Figure 1:**
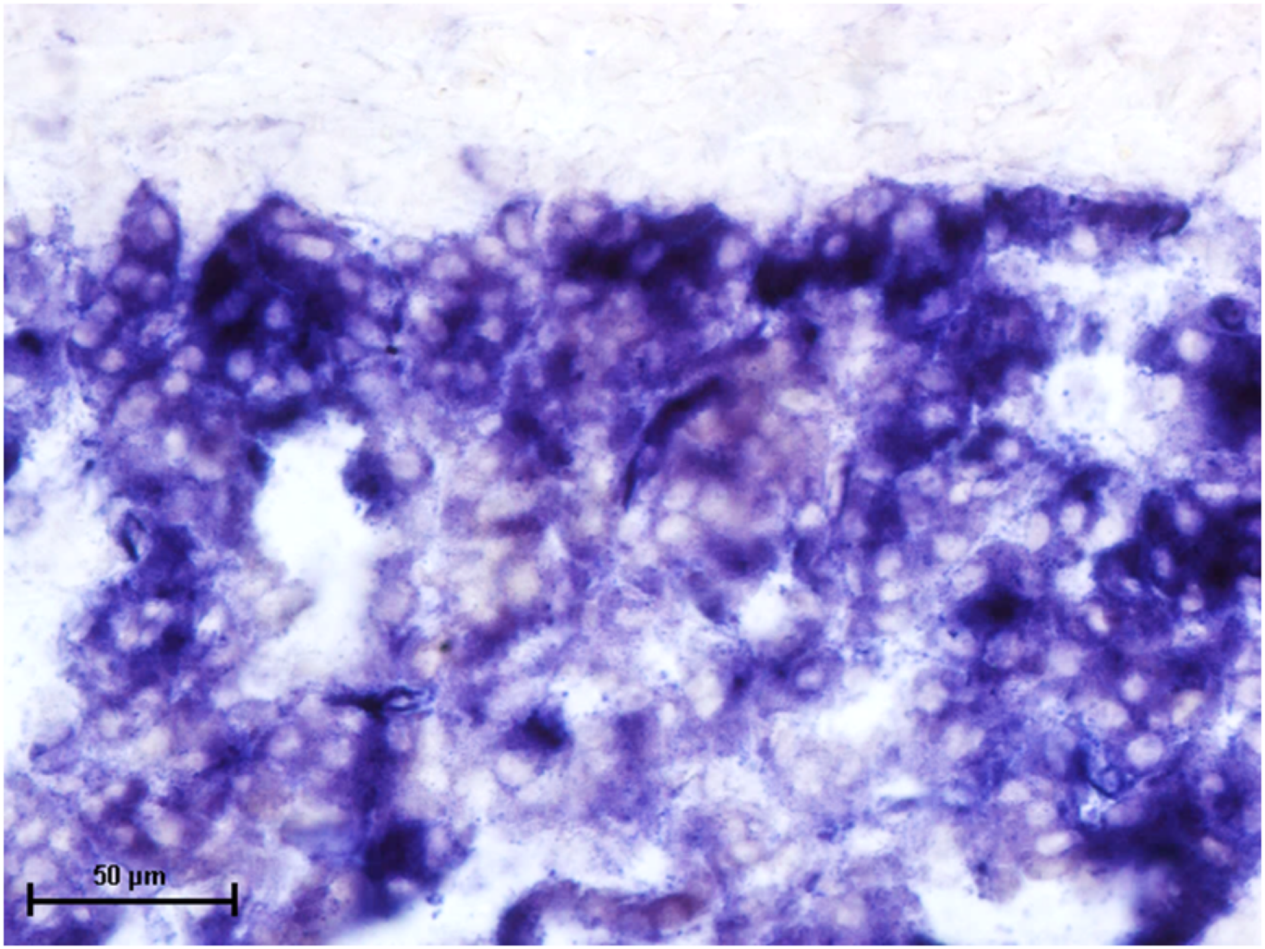
Superficial gland of third eyelid in 90.0 cm CVRL fetus showing strong activity of AKPase in alveoli. Simultaneous coupling azo dye method X 400.

**Figure 2:**
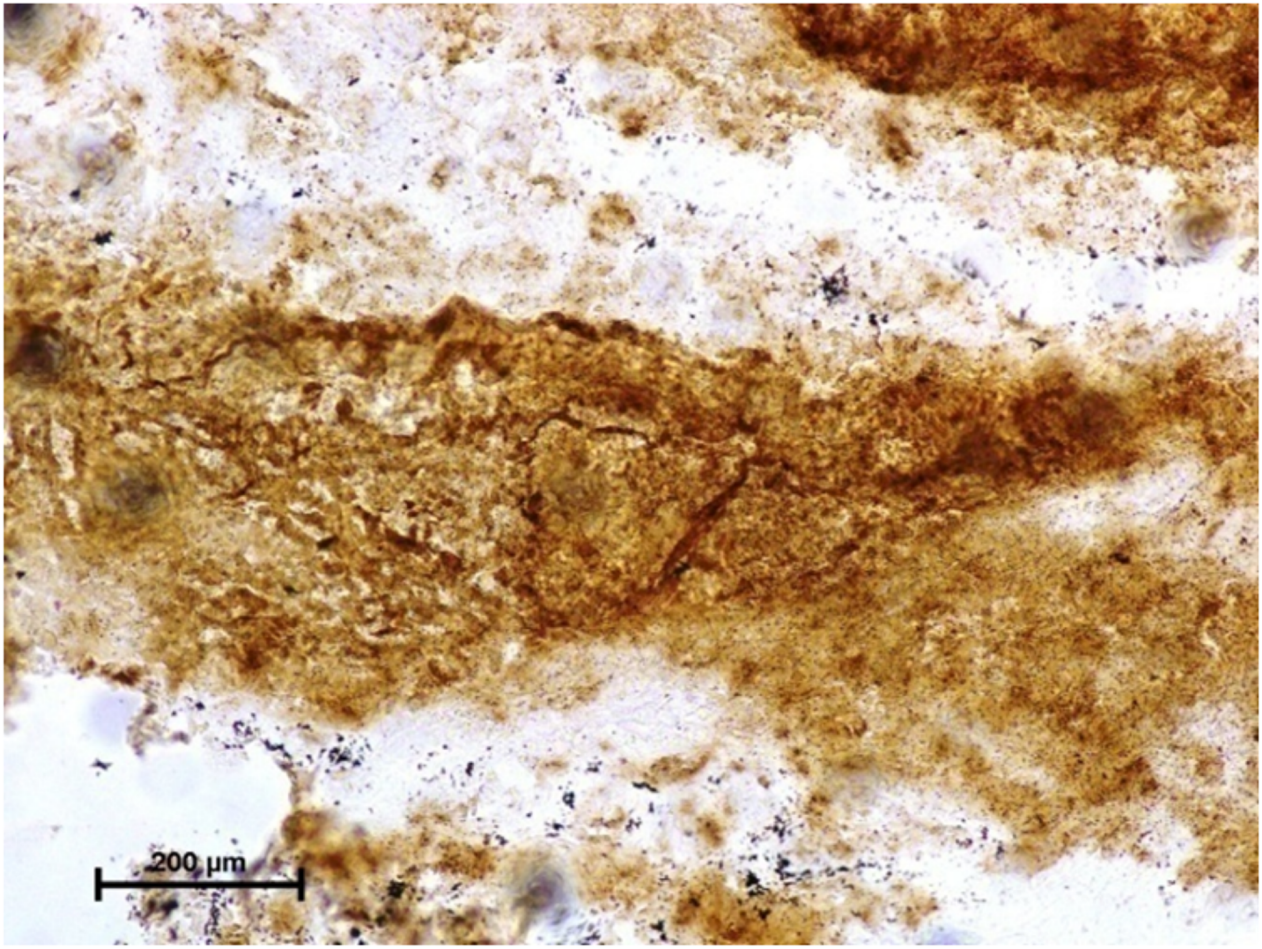
Harderian gland of third eyelid in 92.0 cm CVRL fetus showing strong activity of G-6-Pase in alveoli. Lead nitrate method X 100.

**Figure 3:**
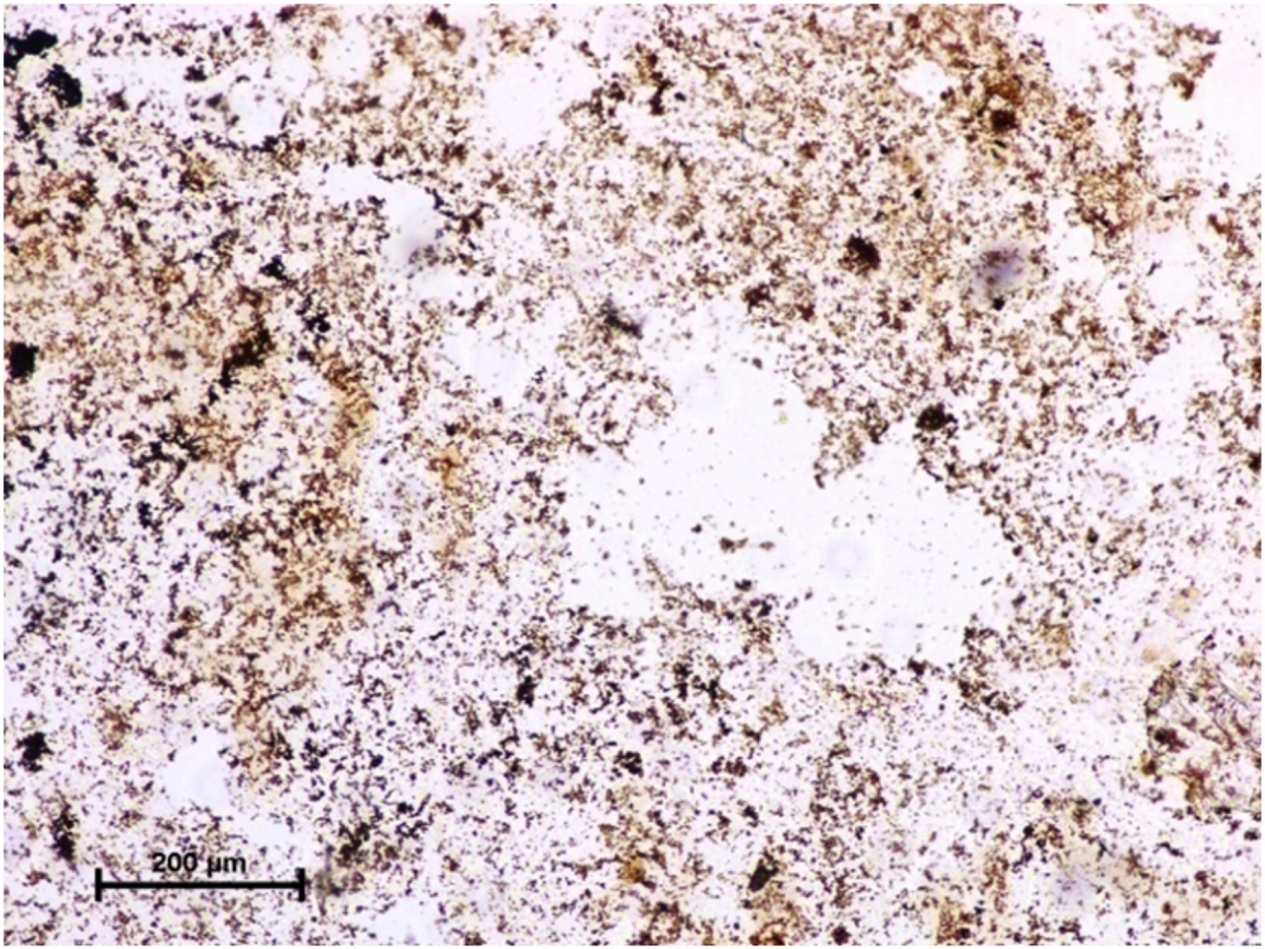
Lacrimal gland of 94.0 cm CVRL fetus showing strong activity of G-6-Pase in alveoli. Lead nitrate method X 100.

Following the incubation for AKPase, the alveoli of all three orbital glands showed strong AKPase activity (Fig. 1). The reaction for G-6-Pase activity was also strong in all three glands (Figs. 2 and 3).

**Figure 4:**
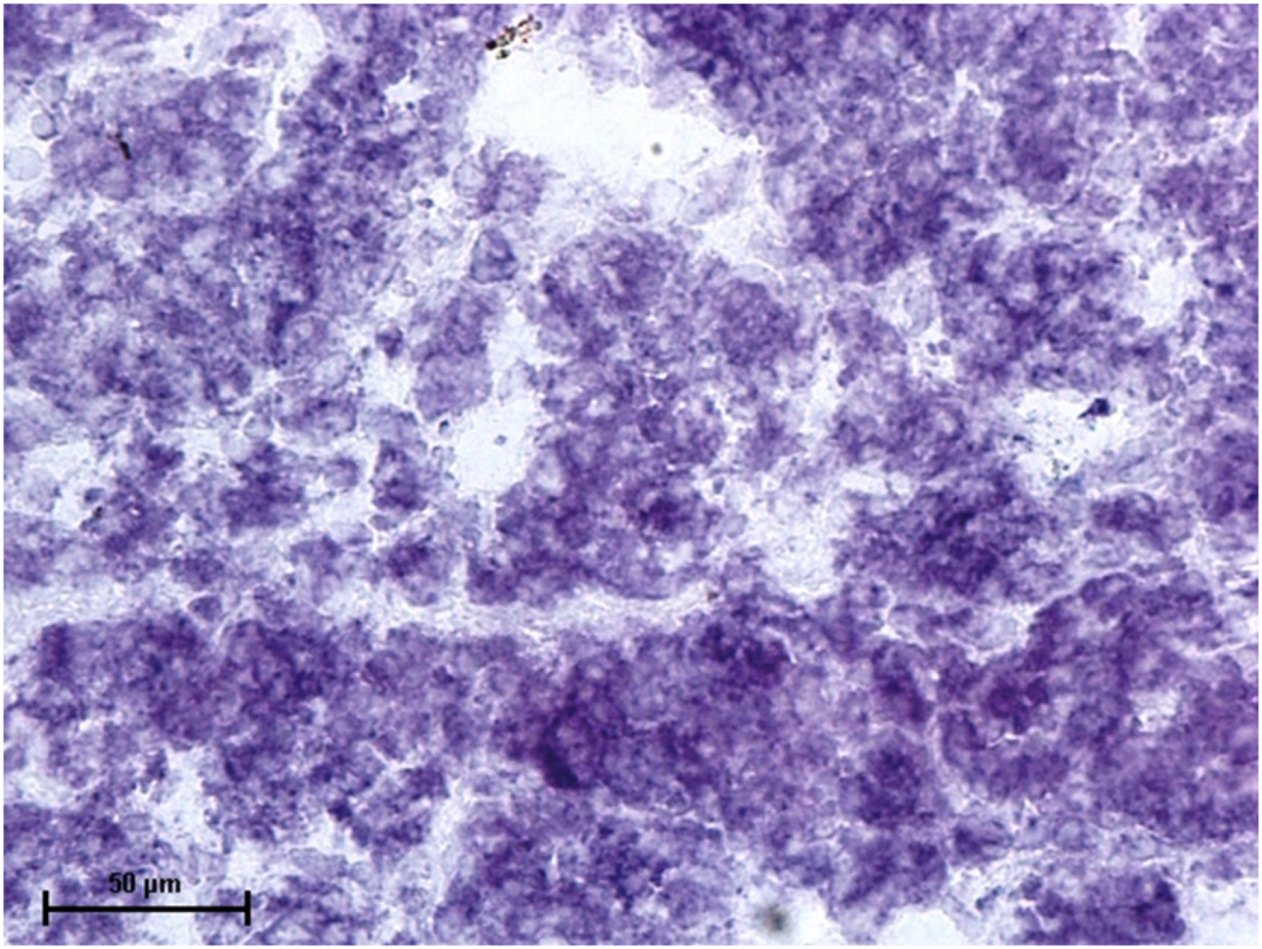
Lacrimal gland of 92.0 cm CVRL fetus showing strong activity of LDH in alveoli. Nitro BT method X 400.

**Figure 5:**
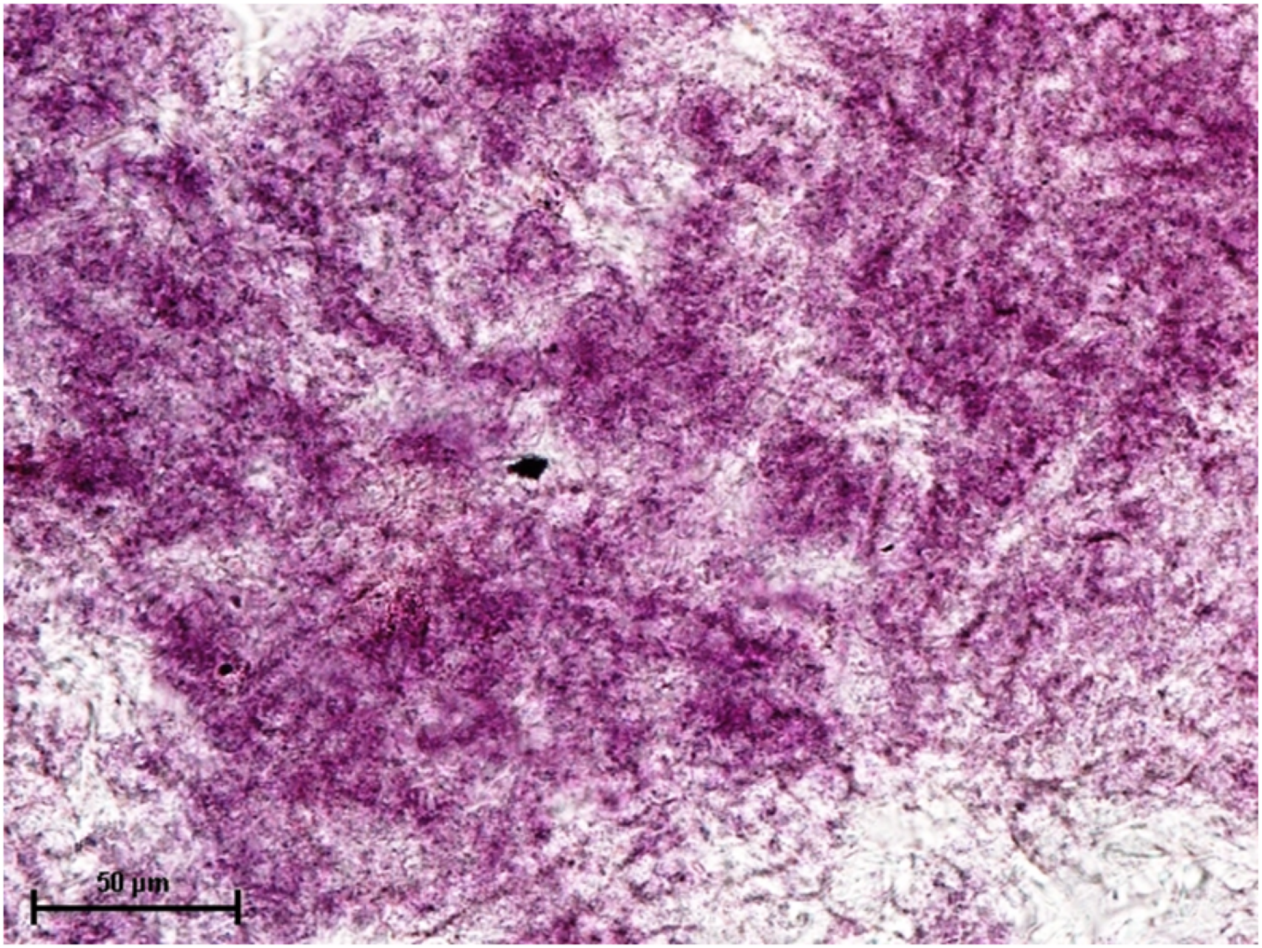
Harderian gland of 90.0 cm CVRL fetus showing strong activity of SDH in alveoli. Nitro BT method X 400.

**Figure 6:**
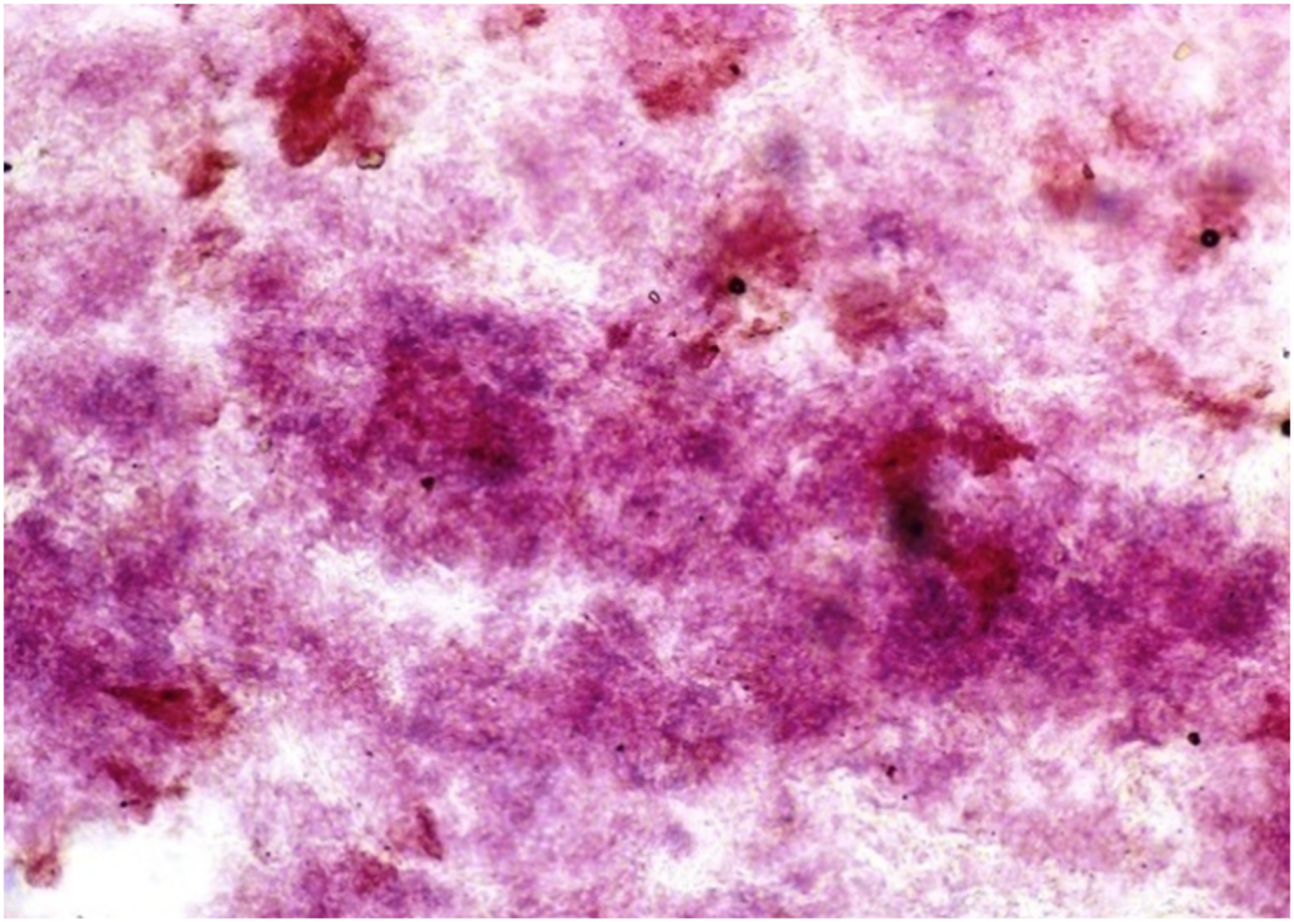
Harderian gland of 92.0 cm CVRL fetus showing strong activity of G-6-PD in alveoli. Nitro BT method X 100.

Under dehydrogenases, cryosections of lacrimal gland showed a strong positive reaction for LDH activity (Fig. 4) whereas the moderate activity was noticed in superficial gland of third eyelid while harderian gland had strong reaction for LDH. The reaction for SDH was found to be strong in all three orbital glands (Fig. 5). Similarly, the reaction for the Glucose-6-phosphate dehydrogenase was also found to be strong in all three orbital glands (Fig. 6).

**Figure 7:**
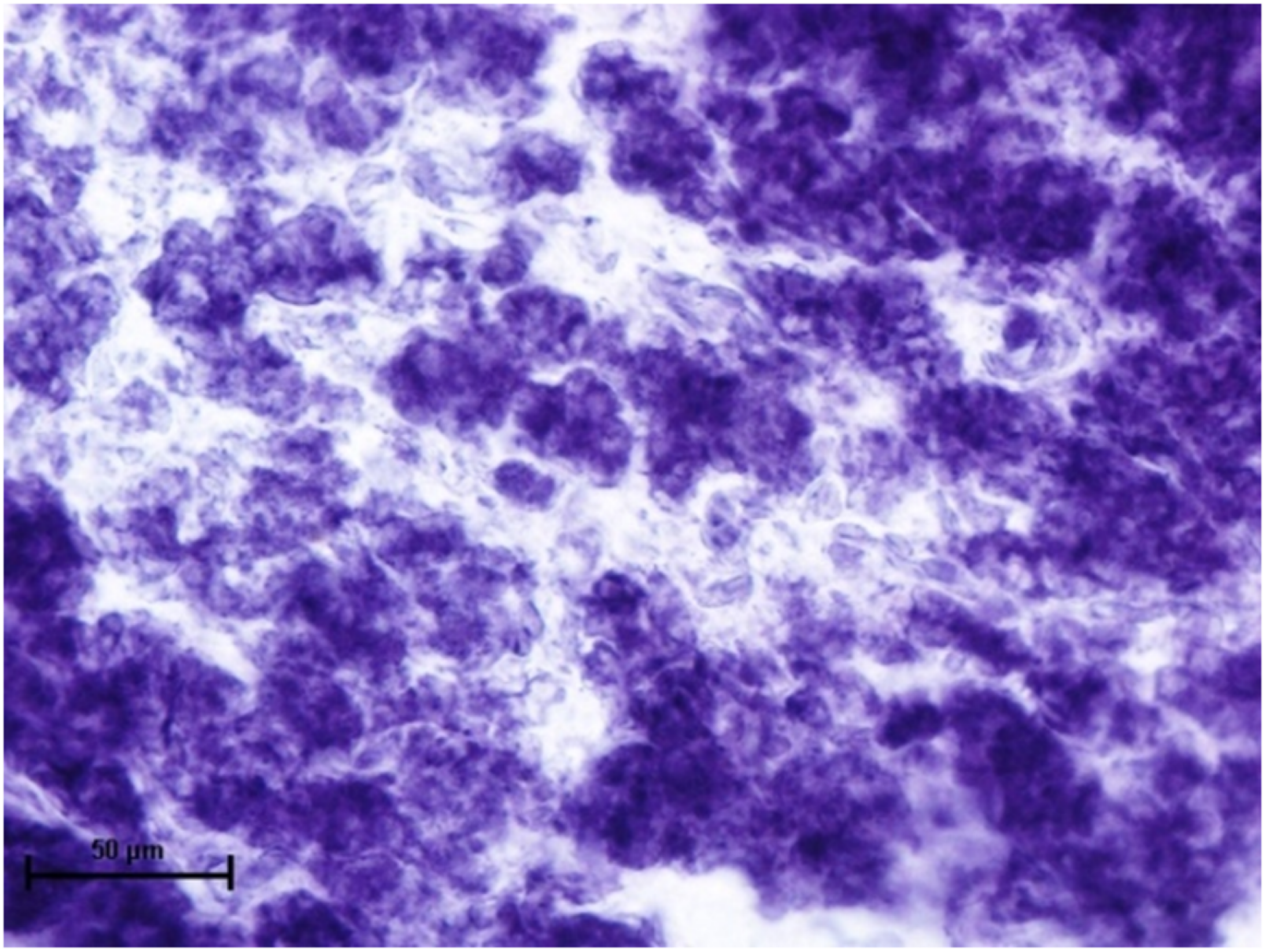
Glandular parenchyma of lacrimal gland in 89.0 cm CVRL fetus showing intense activity of NADHD. Nitro BT method X 400.

**Figure 8:**
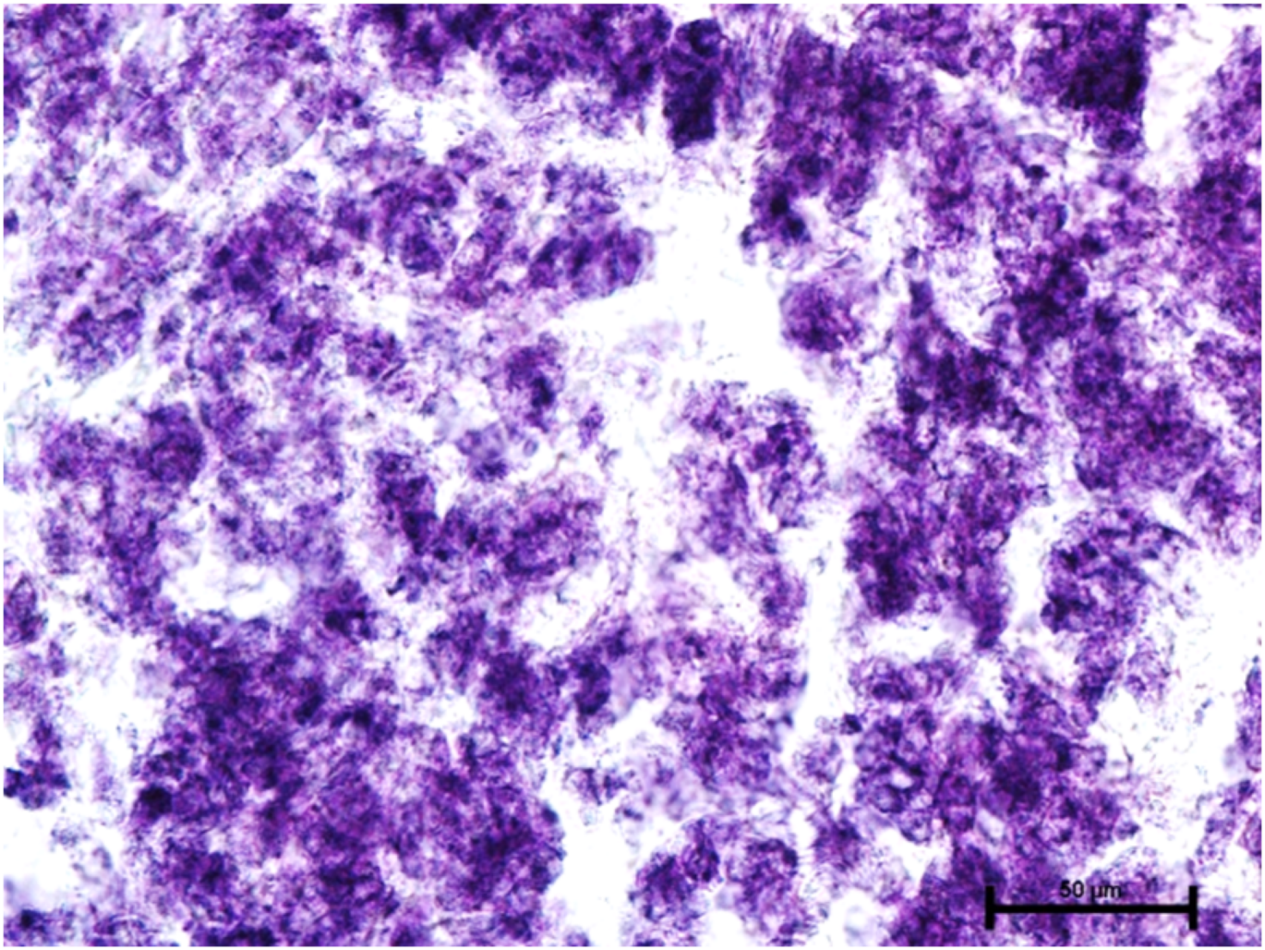
Glandular parenchyma of harderian gland in 92.0 cm CVRL fetus showing intense activity of NADPHD. Nitro BT method X 400.

When these tissues were incubated for the reaction of NADHD, both the glands of third eyelid and lacrimal gland demonstrated intense activity (Fig. 7). The reaction for NADPHD was intense in the lacrimal gland and deep gland of third eyelid while it was strong in superficial gland of third eyelid (Fig. 8).

**Figure 9:**
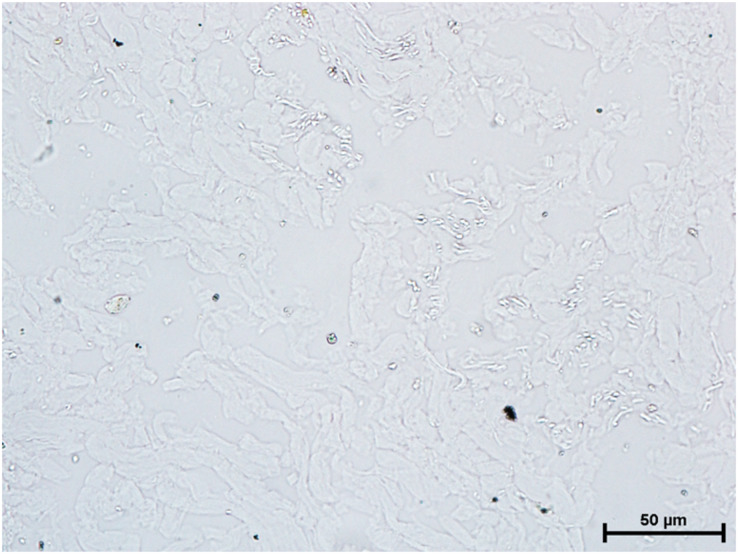
Alveoli of harderian gland in 94.0 cm CVRL fetus showing no activity of DOPA-O. Nitro BT method X 400.

Since, these glands develop in the close vicinity of the eyelids therefore the tissues from these glands were also incubated for the reaction of DOPA-O and tyrosinase activity to evaluate for any reaction or evidence of melanin activity. The results revealed that the activity of both the enzyme was absent in all three orbital glands (Fig. 9).

**Figure 10:**
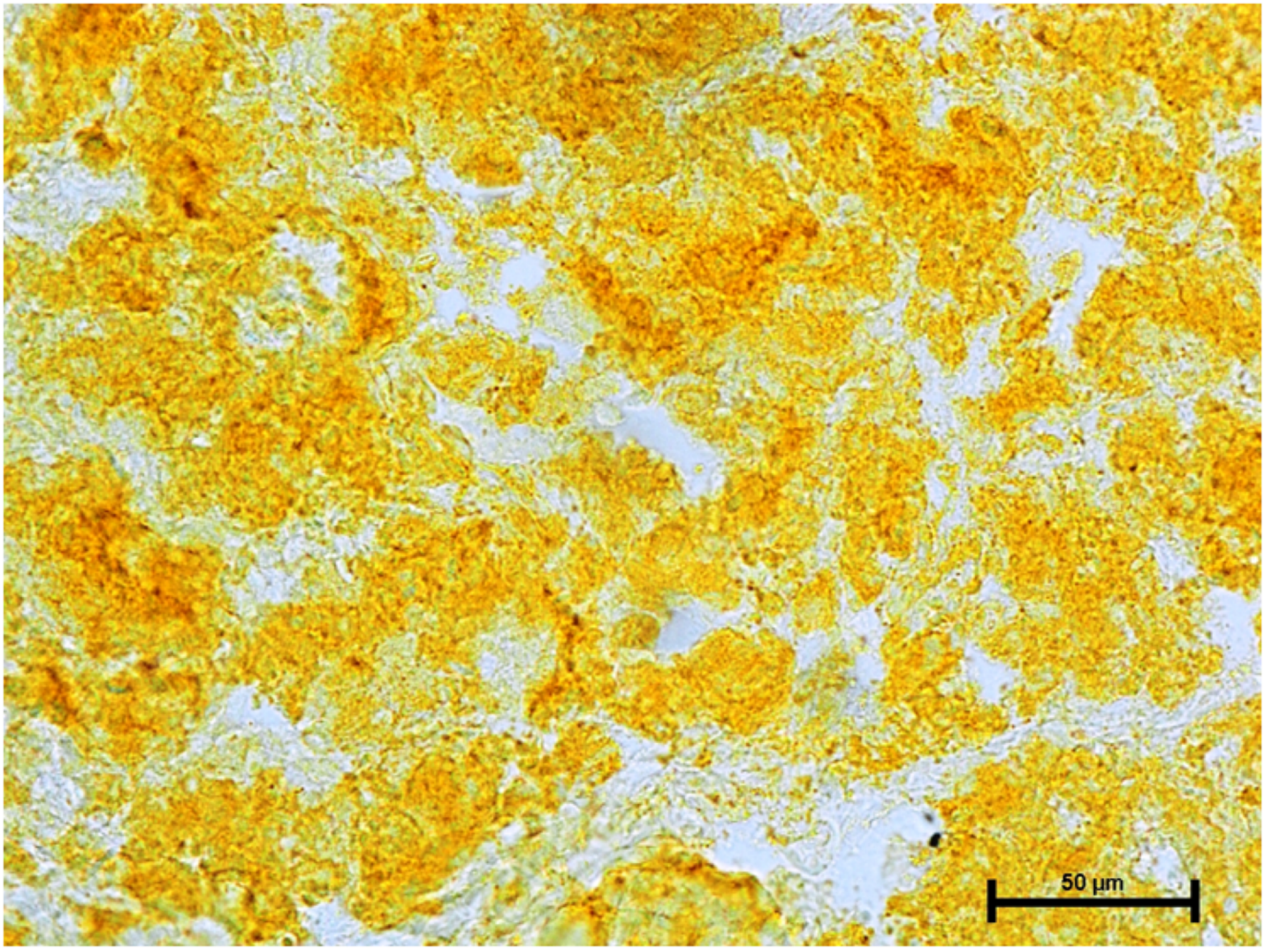
Superficial gland of 90.0.cm CVRL fetus showing strong activity of NSE. X 400.

Incubation for activity of nonspecific esterase resulted in strong activity of the nonspecific esterase in all three glands (Fig. 10).

**Figure 11:**
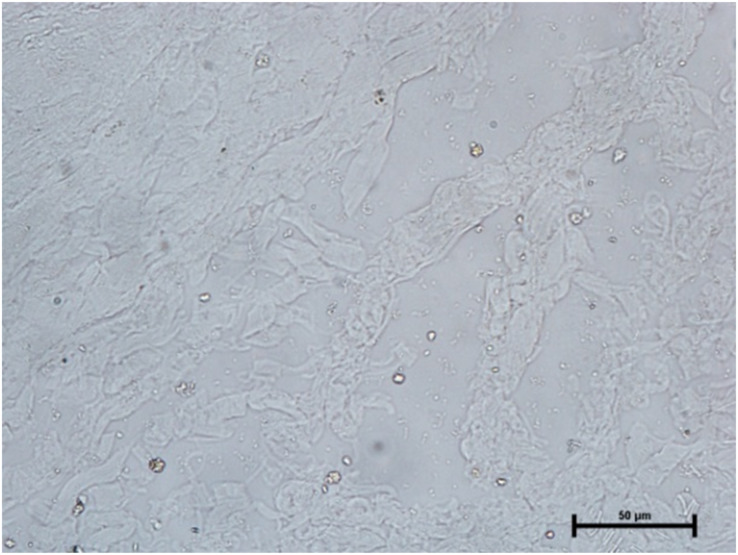
Negative reaction for CAse in lacrimal gland of 89.0 cm CVRL fetus. X 400.

The activity of carbonic anhydrase could not be seen in any of these three glands (Fig. 11). The observations of the enzymatic activity in orbital glands are summarized in Table 1.

**Table 1:** The activity of various enzymes (based on grades of color intensity) in lacrimal gland, superficial and deep gland of third eyelid.

Enzyme	LG	SGT	HG
AKPase	+++	+++	+++
G-6-Pase	+++	+++	+++
LDH	+++	++	+++
SDH	+++	+++	+++
G-6-PD	+++	+++	+++
NADHD	++++	++++	++++
NADPHD	++++	+++	++++
NSE	+++	+++	+++
DOPA-O/Tyrosinase	−−	−−	−−
CAse	−−	−−	−−

**Notes:**

−− for negative, + for weak positive, ++ for moderate positive and +++ for strong positive reactions.

− = negative, 0/+ = negligible to weak positive, + = weak positive, ++ = moderate, +++ = strong, ++++ = intense.

## Discussion

The strong activity of AKPase and G-6-Pase was observed in alveoli of all three orbital glands in present study. However, a weak reaction of AKPase was reported in the alveoli and their ducts in lacrimal gland of neonatal buffalo calves, while a moderate to strong reaction for G-6-Pase was also observed in the alveoli of lacrimal gland in neonatal buffalo calves (Singh, Malhi & Singh, 1999). The AKPase catalyzes the processes of synthesis and transport of substances across the cell membrane and required for the growth, proliferation and differentiation of fetal cells whereas the glucose-6-phosphatase enzyme is responsible for glucose and steroid metabolism (Nelson & Cox, 2004). The strong activities of these enzymes were suggestive of higher metabolic activity in these glands of buffalo fetuses, although the weak reaction of AKPase enzyme in lacrimal gland of buffalo calf indicates that the cellular proliferation and differentiation was inhibited after birth and shows the completion of differentiation during fetal life only (Singh, Malhi & Singh, 1999).

The activity of LDH, SDH and G-6-PD was strong in lacrimal and deep gland of third eyelid but LDH activity was moderate in superficial gland of third eyelid of buffalo fetus. These enzymes are the important catalysts in oxidative metabolism and are involved in Krebs’s cycle. The role of G-6-PD was also linked with high activity of cellular proliferation and differentiation (Ganea & Harding, 2006). A presence of LDH was also noticed in the tear and the fluid of lacrimal gland of rat and rabbit (Thorig, Van Haeringen & Wijngaards, 1984). Similarly, a moderate to strong reaction was also reported in the acini of lacrimal gland in neonatal buffalo calves (Singh, Malhi & Singh, 1999). The involvement and high activity of G-6-PD was also noticed during corneal epithelial growth and cell differentiation increased up to 150-fold when corneal epithelial cells constituted a differentiated four- to five-layered epithelium. It was also observed that the specific activity of glucose-6-phosphate dehydrogenase declined with age in the whole rat lens, however this decline was non-significant (Ganea & Harding, 2006). Therefore, it can be postulated that the strong activity of these enzymes in fetal orbital glands play a significant role in metabolism, cell proliferation and differentiation.

The glands of third eyelid and lacrimal gland demonstrated intense activity for NADHD but for NADPHD, the lacrimal and superficial glands of third eyelid showed intense reaction while deep glands showed strong activity. Earlier, a moderate to strong activity of NADHD was noticed in the acini of lacrimal gland in neonatal buffalo calves (Singh, Malhi & Singh, 1999). The similar activity of NADPHD was noticed in the lacrimal gland of neonatal buffalo calf (Singh, Malhi & Singh, 1999). Diaphorase enzymes are a crucial part of the electron transport chain in aerobic energy production. This energy is used for a variety of cellular processes, such as the synthesis of different substrates, growth, proliferation, contraction of muscles, transmission of nerve impulses, and secretion from glandular tissue. Hence, the functional state of numerous cells in the developing buffalo eye and its appendages throughout prenatal life may be related to the spectrum of activity for this category of diaphorase. The present study’s strong to severe diaphorase activity may be associated to a greater foetal energy metabolism.

The orbital glands have a close association with the eyelids which have melanin therefore, the orbital gland tissues were checked for the enzyme related to melanin synthesis *viz*. DOPA-O and Tyrosinase. However, any activity of these enzymes could not be observed in any of the orbital gland which indicated the absence of any component of melanin synthesis. The lacrimal gland had intense activity for the nonspecific esterase which showed the active involvement of lipid metabolism in the gland.

The presence of bicarbonates in the tear was already reported. The tears are secreted from orbital glands especially from the lacrimal gland (Davidson & Kuonen, 2004). Therefore, it was hypothesized that the activity of enzymes related to bicarbonate metabolism must be higher in orbital glands. The most abundant enzyme for bicarbonate metabolism is carbonic anhydrase (Nelson & Cox, 2004). But when the tissues from orbital glands were subjected to the incubation for CAse, no activity was reported in any orbital gland in present investigation. This finding caught us off guard and was later supported by reports stating that neonates begin to generate tears from the age of two weeks after birth and that they can be marked between one and three months of age (Bradley, 2020). Similarly, the activity of carbonic anhydrase was also observed in some components in post-natal rabbit eye (Drecoll & Lonnerholm, 1981). Therefore, the absence of CAse activity in fetal orbital glands indicated that there may not be bicarbonate metabolism in orbital glands of buffalo fetuses, which also showed that there may not be any tear formations in the fetal eyes of buffalo.

## Conclusions

From the present investigation, it can be concluded that the cellular proliferation, differentiation and growth were the prominent factors in fetal orbital glands of buffalo for which the activities of enzymes related to energy metabolism, phosphate transfers between the molecules and lipid metabolism were high. Also, even being in close association with the eyelids, the activity of enzymes related to melanin synthesis was not witnessed. The absence of activity of CAse showed that bicarbonate metabolism may not be present during fetal life and that justifies that the tear formation starts after birth only.

## Additional Information and Declarations

### Data Availability

The following information was supplied regarding data availability: The scores of reactivity for various enzymes are available in Table 1.
